# Lutetium-177 DOTATATE Production with an Automated Radiopharmaceutical Synthesis System

**Published:** 2015

**Authors:** Alireza Aslani, Graeme M Snowdon, Dale L Bailey, Geoffrey P Schembri, Elizabeth A Bailey, Nick Pavlakis, Paul J Roach

**Affiliations:** 1 Department of Nuclear Medicine, Royal North Shore Hospital, Sydney, Australia; 2 Sydney Medical School, University of Sydney, Sydney, Australia; 3 Faculty of Health Sciences, University of Sydney, Sydney, Australia; 4 Department of Medical Oncology, Royal North Shore Hospital, Sydney, Australia

**Keywords:** Automated synthesis, Lutetium-DOTATATE, Neuroendocrine tumours, Peptide Receptor Radionuclide Therapy

## Abstract

**Objective(s):**

Peptide Receptor Radionuclide Therapy (PRRT) with yttrium-90 (^90^Y) and lutetium-177 (^177^Lu)-labelled SST analogues are now therapy option for patients who have failed to respond to conventional medical therapy. In-house production with automated PRRT synthesis systems have clear advantages over manual methods resulting in increasing use in hospital-based radiopharmacies. We report on our one year experience with an automated radiopharmaceutical synthesis system.

**Methods:**

All syntheses were carried out using the Eckert & Ziegler Eurotope’s Modular-Lab Pharm Tracer® automated synthesis system. All materials and methods used were followed as instructed by the manufacturer of the system (Eckert & Ziegler Eurotope, Berlin, Germany). Sterile, GMP-certified, no-carrier added (NCA) ^177^Lu was used with GMP-certified peptide. An audit trail was also produced and saved by the system. The quality of the final product was assessed after each synthesis by ITLC-SG and HPLC methods.

**Results:**

A total of 17 [^177^Lu]-DOTATATE syntheses were performed between August 2013 and December 2014. The amount of radioactive [^177^Lu]-DOTATATE produced by each synthesis varied between 10-40 GBq and was dependant on the number of patients being treated on a given day. Thirteen individuals received a total of 37 individual treatment administrations in this period. There were no issues and failures with the system or the synthesis cassettes. The average radiochemical purity as determined by ITLC was above 99% (99.8 ± 0.05%) and the average radiochemical purity as determined by HPLC technique was above 97% (97.3 ± 1.5%) for this period.

**Conclusions:**

The automated synthesis of [^177^Lu]-DOTATATE using Eckert & Ziegler Eurotope’s Modular-Lab Pharm Tracer® system is a robust, convenient and high yield approach to the radiolabelling of DOTATATE peptide benefiting from the use of NCA ^177^Lu and almost negligible radiation exposure of the operators.

## Introduction

Neuroendocrine tumours (NETs) comprise a wide spectrum of tumours arising from neural crest cells throughout the body. While once considered rare, recent epidemiologic data has shown increasing incidence with little change in mortality, suggesting that there has been significant under-diagnosis in the past (1). These tumours are often slow growing and may go undiagnosed for long periods of time leading to patients presenting with advanced disease. Symptoms are frequently vague leading to delays in diagnosis. The appropriate management of these tumours is best handled through a multidisciplinary approach as their diagnosis and treatment crosses a range of specialities.

Neuroendocrine gastrointestinal tumours are the most common. Terminology has been varied over the years and these are often broken down into carcinoid (mid-gut) tumours and endocrine pancreatic neoplasms. While these two groups have different behaviour, they are often encompassed in the single term - gastroenteropancreatic neuroendocrine tumours (GEP-NETs). Many of these tumours (the so called carcinoid tumours) produce endocrine syndromes due to the overproduction of a range of hormones.

The fact that NETs overexpress subtypes of somatostatin (SST) receptors (2) allows for diagnosis and treatment with SST analogues. While all five SST receptor subtypes are overexpressed to some degree (3), SST_2_ is the most widely overexpressed. Peptide Receptor Radionuclide Therapy (PRRT) has gained substantial interest in the last few years (4). Radiolabelled somatostatin analogues are now considered for patients who have failed to respond to conventional medical therapy (5, 6). The two most commonly used radioisotopes are yttrium-90 (^90^Y) and lutetium-177 (^177^Lu). These radioisotopes differ in terms of their emitted radiations, particle energy, physical half-life and tissue penetration (7, 8). The radiopharmaceuticals being used for this therapy are DOTATATE, DOTATOC and DOTANOC, all of which are labelled via the DOTA chelator to either ^90^Y or ^177^Lu (7). These radiopharmaceuticals have a range of SST affinities though all have good affinity for SST_2_. Although the synthesis and purification of these radiopharmaceuticals can successfully be carried out manually in the laboratory, the use of automated synthesis systems is gradually increasing (9). These automated systems have clear advantages over manual methods which have resulted in their increasing installation and use in hospital-based radiopharmacies. At our institution we use no-carrier added (NCA) ^177^Lu labelled with a locally synthesised peptide for the PRRT of patients with well-differentiated NETs. The [^177^Lu]-DOTATATE (referred to as “NCA-LuTATE”) is synthesised in-house using an automated synthesis system. Here we report on our one year, 17 syntheses experience with this formulation.

## Methods

There are a number of reports in the literature using and detailing various automated radiochemistry systems used for synthesising radio-pharmaceuticals (RP) labelled with gallium-68 (^68^Ga), ^177^Lu and other radioisotopes (10-12). However, the number of reports in the literature using automated synthesis systems for [^177^Lu]-DOTATATE is scarce except for de Decker and Turner (13) who recently reported their experience of automated synthesis systems for [^177^Lu]-DOTATATE synthesis. Automated RP synthesis systems use generic synthesis procedures with minor modifications tailored to the specific needs of the institution and can also include chemical analysis and detection systems such as High Pressure Liquid Chromatography (HPLC) units. As a result, over time, these setups evolve and are modified to improve productivity and can become unique to the institution. The system reported here was the Eckert & Ziegler Eurotope’s Modular-Lab Pharm Tracer® automated synthesis system.

All materials and methods used were followed as initially instructed or later modified by the manufacturer of the system (Eckert & Ziegler Eurotope, Berlin, Germany - subsequently referred to as the vendor or manufacturer) from whom the automated RP synthesis system and synthesis cassettes were obtained.

### 

#### Reagents

As recommended by the manufacturer, all reagents were high purity pharmaceutical grade, unless stated otherwise. The exact grade as well as the source of the reagents used was also their recommendation.

Water puriss p.a (FLUKA Trace SELECT® 95305) used in the preparation of the majority of reagents was obtained from Sigma-Aldrich, Australia. Sodium chloride 0.9% for injection in 50 mL glass bottles (INJ089) were obtained from Phebra (Lane Cove, NSW, Australia). The L-ascorbic acid puriss p.a (FLUKA 33034-100 G), sodium hydroxide monohydrate (Trace SELECT® 01968-25 GF; ≥99.9995% pure), and 200 proof ethanol (HPLC / spectrophotometric grade, 459828-1L) were also obtained from Sigma-Aldrich, Australia.

^177^Lu was produced by a different method to that commonly reported previously. Two distinct methods for reactor production of ^177^Lu can be used: the more commonly used approach is to directly irradiate high purity ^176^Lu via the reaction ^176^Lu(n,γ)^177^Lu, while the alternative is to irradiate ^176^Yb as in ^176^Yb(n,γ)^177^Yb which decays with a 1.9 hour half-life to ^177^Lu. The latter method has the advantages that (a) there is no carrier lutetium present, and so is referred to as “no-carrier added” lutetium (NCA [^177^Lu]), and (b) there is no ^177m^Lu produced, which has a physical half-life of 160 days and therefore presents a significant radioactive waste storage and disposal problem. A further advantage of the NCA [^177^Lu] is that it requires less peptide to label the necessary active amount of [^177^Lu]-DOTATATE for the therapy. This is the production methodology for the ^177^Lu utilised at our institution.

The DOTATATE, or DOTA-(Tyr^3^)-octreotate (where DOTA = 1,4,7,10-tetraazacyclododecane– 1,4,7, 10-tetraacetic acid) was obtained from Auspep (Melbourne, Australia). The peptide is provided as a 1 mg powder which is subsequently dissolved in 1 mL of water and used in 200 µL aliquots. The DOTATATE peptide is certified as Good Manufacturing Practice (GMP) grade.

The preparation of the required [^177^Lu]-DOTATATE reagents and chemicals are shown in Table 1. This table is used as a quick instruction guide for the preparation of chemicals at our centre.

**Table 1:** Checklist of chemicals used for [^177^Lu]-DOTATATE synthesis

Name of Solution	Preparation	Product Code
	Prepare 2 solutions	Ascorbic Acid, Sigma Aldrich 33034-100 G
	Solution 1: Dissolve 2.2 g ascorbic acid in 22 mL water (puriss p.a.)	Sodium hydroxide, Sigma Aldrich 01968-25 G-F
	Solution 2: Dissolve 0.9 g sodium hydroxide in 2.1 mL water (puriss p.a.)	Water Puriss. p.a., FLUKA 95305
	Adjust pH to 4.5± 0.1:	
Ascorbic Acid Buffer stock solution (0.57 M, pH 4.5)	Add 1 mL of solution 2 to solution 1. Use 0.5 mL sample to check pH. Discard sample after measurement. Add more solution 2 in small steps and measure pH of samples (0.5 mL). Discard samples after measurement.	
	Stop adjusting at pH to 4.5± 0.1.	
	Inject buffer samples through sterile filter (0.2 µm) aliquots into dry sterile vials.	
	Radiolabelling vol­ume: 2-5 times high volume Ascorbic buffer with respect to radioactivity volume.	
	Store at -20 °C not longer than 1 month	
Ethanol solution	9.5 mL ethanol	Ethanol 200 proof HPLC/Spectrophotometric grade Sigma Aldrich 459828-1L
	10.5 mL H_2_O	Water Puriss. p.a., FLUKA 95305
NaCl (saline)	50 mL sterile saline	Sodium Chloride 0.9% Injection 900 mg in 50 mL Phebra INJ072
DOTATATE	Stock DOTATATE XX mg peptide in XX mL H_2_O;	DOTA-(TYR3) Octreotate acetate salt 2500437 1 mg CAS-No 177943-89-4
	Optimum conditions for ^177^Lu labelling: 1 µg DOTA peptide per 40 MBq of ^177^Lu.	Water Puriss. p.a., FLUKA 95305
	e.g., 8 GBq dose: 200 µg DOTA peptide. Store in freezer	
	Dispense 200 mL into eppendorf vials. Store in freezer	
[^177^Lu]-DOTATATE ITLC QC	0.1 M Na Citrate pH 5.0 100 mL	

The ^177^Lu (NCA ^177^LuCl_3_) was obtained from ITG (Isotope Technologies Garching GmbH, Germany) through a partnership with the Australian Nuclear Science and Technology Organisation (ANSTO), Sydney, Australia. The amount of radioactive ^177^Lu ordered for each synthesis was based on the number of patients to be treated per synthesis and an estimate of 80% synthesis yield. The prescribed dose to be injected was a notional 8 GBq per patient (14).

#### Synthesis Cassettes and Automation

The synthesis of [^177^Lu]-DOTATATE can be separated into three parts. These are:

a) the modules, arranged according to the specific RP being synthesised;
b) the synthesis cassettes on which the appropriate chemicals, reagents, filters, columns, etc are attached; and
c) the computer system that executes the software which, in turn, operates the valves and syringes on the cassettes producing the required RP.

Since the system is computer-controlled, an audit trail is always maintained which is a requirement for GMP production certification. There is a specific synthesis cassette for the production of [^177^Lu]-DOTATATE. The same system is also currently being used for the production of other PET RP, such as [^68^Ga]-DOTATATE and [^68^Ga]-PSMA (Prostate-Specific Membrane Antigen) using separate synthesis cassettes as well as another for a simple elution of the ^68^Ge/^68^Ga generator. All production cassettes are obtained from the vendor. These cassettes are sterile and for single-use only.

As with the cassettes, there is a specific computer template program for each RP synthesis process. These templates are also provided by the vendor. These are subdivided into programs for the cassette pressure testing, terminal sterilisation filter testing, running the HPLC analysis and, in the case of [^68^Ga]-DOTATATE and [^68^Ga]-PSMA, also for eluting the ^68^Ge/^68^Ga generator. The software also allows programming and modifications to the templates via a graphical user interface (GUI).

#### Lutetium-177 (^177^Lu)

Lutetium-177 (t_½_ = 6.7 days) emits both β^-^ and γ radiation during its decay to stable ^177^Hf. The principal β^-^ emission has an average energy of 0.149 MeV with 78.6% abundance. There are a number of small abundance γ photons emitted, with those of interest for imaging using the gamma camera being 0.208 MeV (11% abundance) and 0.113 MeV (6% abundance). The fact that few γ photons are emitted allows many ^177^Lu therapies to be performed on an outpatient basis from a radiation safety perspective in many jurisdictions despite the patient being administered up to 8-10 GBq of ^177^Lu, with exposure from contact with treated patients being comparable to that seen with a ^99m^Tc bone scan. The ^177^Lu we have used for all syntheses was sterile, GMP-certified, no-carrier added ^177^Lu in the form of lutetium chloride (^177^LuCl_3_) and is supplied in a volume of 0.5 mL (0.04 M HCl solution). The radioisotope generally arrives at our centre one day prior to the actual day of synthesis. The radioactivity was calibrated for the day and time the synthesis was due to be carried out. The radioactivity ordered was requested to be approximately 10 GBq per patient which allowed for an approximate 80% product yield providing a final 8 GBq dose per patient. To date, up to four patient doses have been produced in a single synthesis, i.e., an approximate amount of 40 GBq of NCA ^177^Lu at start of synthesis.

#### Automated Radiolabelling Process

**Figure 1:**
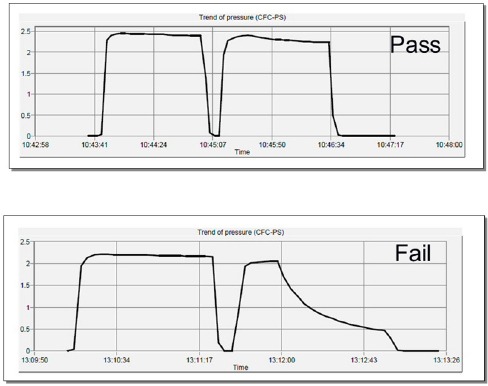
Pressure test trace indicating a pressure test Pass (top) and a Fail (bottom)

Once the sterile synthesis cassette is removed from its packaging and all connections tightened, it is attached to the Modular Lab’s synthesis cassette module. The radiolabelling process is performed in three steps. The first step tests the cassette for any leaks. This step, also known as the Cassette Pressure Test, applies a pressure of 200 kPa to various sections of the cassette. This is software controlled. This test is performed by attaching the cassette to a high purity nitrogen gas supply with its pressure regulator adjusted to deliver 200 kPa of pressure. A visual progress graph is also displayed (Figure 1). If there is no loss of pressure to below 100 kPa, the test is considered to have passed and the cassette is suitable for use. However, if the test fails, the cassette is removed from the module, all connections re-tightened and test repeated. If the test fails again, the cassette is rejected and sent back to the manufacturer and replaced. The process of pressure testing the cassette takes approximately five minutes. On completion, the cassette pressure test process log is saved on the computer for audit trail purposes. On passing the test, the necessary pre-prepared reagents are placed in the appropriate containers of the synthesis cassette and ^177^Lu vial connected. The empty containers, the waste bottle and product vial are then attached. The product vial and waste bottle are placed in a specially designed Perspex container boxes to reduce any β^-^ radiation dose to the operator. The vial containing the ^177^Lu is kept in its original lead shielded container also to reduce radiation dose to the operator. The second step of the synthesis phase is similarly driven by the Modular Lab Pharm Tracer® software. The duration of this process is approximately 45 minutes and is the main step of the entire process. The movement of all liquids is driven by a 10 mL syringe attached to the syringe-driver module. This phase involves a number of different steps, including washing of various sections with 0.9% saline. Briefly, the Waters tC18 Light reverse phase silica cartridge (also known as C18 ion exchange or SepPak® cartridge) is initially primed (wetted) with ethanol followed by 0.9% saline. The [^177^Lu]-LuCl_3_ is then transferred from its vial (radioactivity vial) into the reactor which contains the DOTATATE peptide. To ensure that the entire radioactivity has been removed from the delivery vial, it is then washed with the buffer solution and the remaining radioactivity is transferred to the reaction vial. Once all the lutetium is in the reaction vial together with the peptide, the mixture is heated at 80° C for 20 min. The cassette is then rinsed twice with 0.9% saline to remove any residual radioactivity from the fluid paths. The crude product from the reaction vial is then removed and kept in the syringe for five minutes to cool. Once cooled, it is then passed through the C18 cartridge to remove any unbound [^177^Lu]-LuCl_3_. Both the C18 cartridge as well as the reaction vial are then rinsed with 0.9% saline to recover any residual product. The product, now trapped in the C18 cartridge, is eluted by first passing an ethanol / water mixture (47.5%:52.5% ratio) and then 0.9% saline into the final product vial. To ensure sterility, the final product is passed through a 0.22 µm filter. The total volume of the final product is 18 mL. The software provides a graphical display of each step and progress of the synthesis. On completion, the synthesis process is digitally saved on the computer for audit purposes.

The third phase of the synthesis involves testing of the integrity of the 0.22 µm filter. This is known as the Filter Pressure Test and is, again, driven by the Modular Lab Pharm Tracer® software. The only operator intervention required is the removal of the 0.22 µm filter and needle from the product vial and putting it into the waste bottle prior to the test. The test is performed by subjecting the filter to 200 kPa of pressure. If pressure is maintained above 100 kPa, the test is considered to have been successful and the product is sterile for patient use. However, if the pressure falls below 100 kPa, the test is considered to have been unsuccessful and there is a chance of the product being unsterile. To rectify this, the product needs to be drawn-up manually in a 20 mL syringe and passed through a new 0.22 µm filter into another sterile vial. To date, this has not occurred at our centre. On completion, the filter pressure test process log is saved on the computer for audit purposes. The trace produced by the software is similar to the ones produced for the Cassette Pressure Test. After removing a 0.2 mL sample from the product for quality control purposes, the product is ready to be administered to the patient.

#### Analysis and Quality Control

The quality control of the resulting product is done by two methods - Instant Thin Layer Chromatography (ITLC) as well as High Pressure Liquid Chromatography (HPLC). These are performed using the 0.2 mL sample taken directly from the final [^177^Lu]-DOTATATE product.

#### Instant Thin Layer Chromatography (ITLC)

The ITLC test is used to determine the percentage of [^177^Lu]-DOTATATE and ^177^Lu impurities in the final product. The ITLC paper strips are counted in a laboratory gamma-counter (PerkinElmer Wizard^2^® Automated Gamma Counter, PerkinElmer Downers Grove, IL, USA) and results entered into a spreadsheet (Microsoft Excel Version 2010) for final calculation and analysis.

For the determination of percentage [^177^Lu]-DOTATATE and ^177^Lu impurities, 0.1 mol/L citrate buffer (pH= 5) with ITLC-SG paper (Pall Corporation, East Hills, New York); R_f_ (free ^177^Lu) = 0.8–1.0, R_f_ (^177^Lu peptide) = 0.0–0.3 is used.

#### High Pressure Liquid Chromatography (HPLC)

The HPLC analyser was provided by the vendor and was driven by the Modular Lab Pharm Tracer® software. The HPLC unit comprises a Knauer Smartline D14163 pump (Knaur, Berlin, Germany) with Knauer Online Degasser V7620 (Knauer, Berlin, Germany), ACE3 C18 column 150×3 mm (Advanced Chromatography Technology, Aberdeen, UK), flow rate 0.6 mL.min^-1^, water / acetonitrile (ACN)/0.1% trifluoroacetic acid with 22% ACN. A 0.04 mL sample is used to determine the percentage [^177^Lu]-DOTATATE content of the final product.

## Results

A total of seventeen [^177^Lu]-DOTATATE syntheses were performed between August 2013 and December 2014 in our laboratory. All syntheses were carried out using the methods described above and were performed in the Department of Nuclear Medicine, Royal North Shore Hospital within a hot-cell (Model: NMCGa-68; TEMA SINERGIE, Italy) located in a purpose-built radiopharmaceutical laboratory. The amount of radioactive [^177^Lu]-DOTATATE produced by each synthesis varied between 10 - 40 GBq and was dependant on the number of patients being treated on a given day.

### Patients

During this period a total of 13 individuals (9 males and 4 females) were treated with [^177^Lu]-DOTATATE on an eight week cycle, equating to a total of 37 individual treatment administrations. All patients were treated on an out-patient basis. Median age at start of treatment for males and females were 60.4 years (range from 32.7 to 70.5 years) and 61.9 years (range from 50.4 to 74.8 years), respectively. The median body surface area (BSA) at start of treatment for males and females were 1.87 m^2^ (range from 1.59 to 2.16 m^2^) and 1.75 m^2^ (range from 1.49 to 1.88 m^2^), respectively.

**Figure 2:**
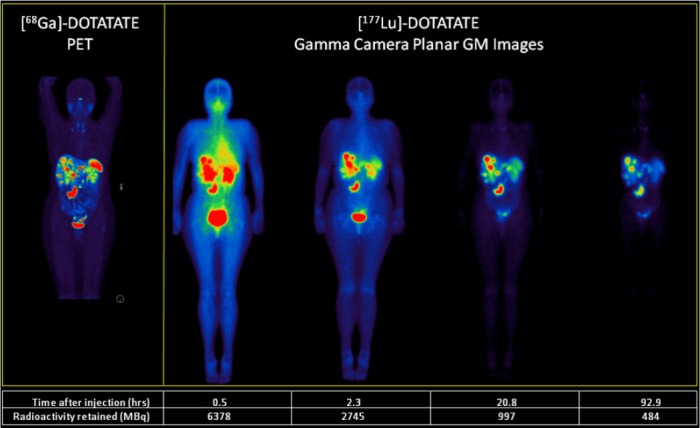
Example of representative images from a single individual showing [^68^Ga]-DOTATATE PET scan (MIP image) (left) and after infusion of ~6.4 GBq of [^177^Lu]-DOTATATE at four time points. The gamma camera images are geometric mean (GM) images that have been corrected for attenuation using a pre-acquired transmission scan and converted to units of radioactivity (kBq) The distribution of disease on [^177^Lu]-DOTATATE images is identical to that seen on the preceding diagnostic [^68^Ga]-DOTATATE scan

Five patients received the full course of 4 therapy cycles during this period. The remaining 8 patients received fewer than 4 cycles either due to being partway through their course of 4 treatments or treatment being suspended due to poor health. An example of the initial biodistribution of the [^177^Lu]-DOTATATE and subsequent retention and biodistribution at a number of time points up to four days post-injection is shown in Figure 2 along with the patient’s corresponding [^68^Ga]-DOTATATE scan. The whole body retention of the radiolabelled peptide averaged over all patients treated to date exhibits a generally bi-exponential pattern with clearance rates of 2.1±0.6 h for the fast and 58.1±7.2 h for the slow components respectively. Approximately 25% of the original amount of administered [^177^Lu]-DOTATATE (approximately 2 GBq) is remaining in the patient when they leave the department approximately four hours after commencing treatment.

The patients were scanned on a SPECT/CT dual head gamma camera (Siemens Symbia T6, Hoffman Estates, IL, USA) using a medium energy collimator (208 keV ±10% PHA window) at 30 min (planar only) and then again at 4, 24, and 96 h (planar and SPECT). The SPECT images were 120 projections at 15 sec per projection at 4 h and 20 sec per projection at 24 and 96 h.

### [^177^Lu]-DOTATATE Syntheses

Although there were no issues and failures with the synthesis cassettes, some minor modifications were made to the volumes of the chemicals, buffers, etc. under the remote guidance of the manufacturer to improve the quality and product yield. These modifications were made as a part of ongoing improvements and quality control in response to user comments and feedback.

### Radiochemical Quality Control

**Figure 3:**
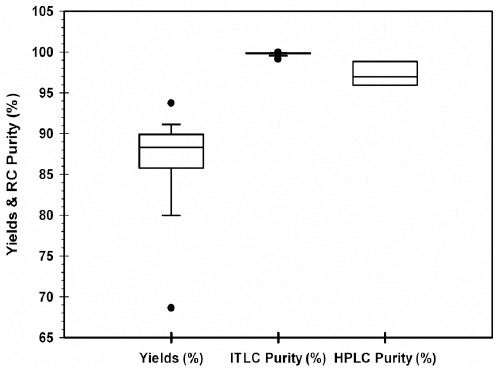
Average [^177^Lu]-DOTATATE yields and radiochemical (RC) purity by ITLC and HPLC

**Figure 4:**
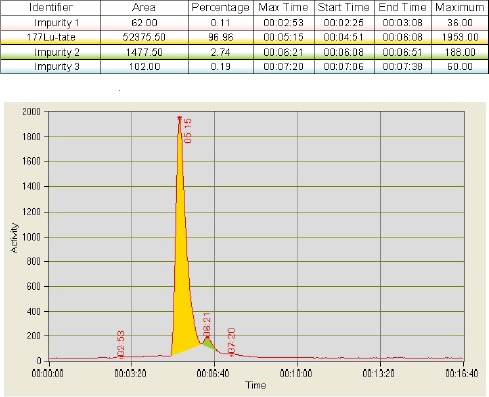
A typical chromatogram report for radiolabelled [^177^Lu]-DOTATATE product

The radiochemical purity of the final product was measured using ITLC as well as HPLC. The use of HPLC for [^177^Lu]-DOTATATE syntheses was initiated recently to have an additional measure of the quality of the final product as well as to compare and experiment on alternative manufacturers’ [^177^Lu]-LuCl_3_. The results demonstrated that the average radiochemical purity as determined by ITLC was above 99% (n=17; 99.8 ± 0.05%) and the average radiochemical purity as determined by HPLC technique was above 97% (n=3; 97.3 ± 1.5%) for this period (Figure 3). A typical chromatogram of the radiolabelled product is shown in Figure 4.

### Cassette and System Failures

As our centre had extensive previous experience with the automated synthesis system for the radiolabelling of [^68^Ga]-DOTATATE (15), the majority of the initial teething problems had already been addressed and rectified. As a result, there have not been any failures during any of the [^177^Lu]-DOTATATE syntheses.

### Record Keeping and Audit Trail

Good record keeping and an audit trail is mandatory for GMP system compliance. For this purpose, electronic copies (in PDF format) of the pressure tests, syntheses, and HPLC tests were automatically saved by the automated synthesis system. In addition, patients’ details, quality control results (ITLC and HPLC), system failures, and synthesis and pressure test batch numbers were recorded in a laboratory log book. This was predominantly for fast access to previous work. A summary of the above was also recorded on a spread sheet (Microsoft Excel Version 2010) and electronically saved on the synthesis PC as well as a hardcopy printed and stored at the Department of Nuclear Medicine.

## Discussion

There has been an increase in the development of targeted radionuclide therapies, especially for cancer. The main benefit observed so far with peptide targeted therapies labelled with β-emitting radionuclides is to stabilize and delay further tumour growth rather inducing significant tumour shrinkage (response). However treatment can improve pain control as well as reduce hormone related symptoms, leading to improved quality of life.

Prior to the wide availability of the automated synthesis systems, the synthesis and preparation of [^90^Y]-DOTATATE or [^177^Lu]-DOTATATE was carried out using “bench-top” manual methods (9). Although there are still centres that use manual methods, there is a move towards the use of automated synthesis systems. Benefits of the automated system include reduced radiation exposure to the operators. Using the manual method and assuming that 400 doses of 7.4 GBq are prepared by four workers, the average equivalent dose to the worker has been reported (16) to be 23 ± 11 mSv and 14 ± 6 mSv for finger top and ring dose, respectively. With the automated synthesis system the finger doses and other exposure is negligible.

In terms of their final product, both synthesis methods are similar, but not identical. One difference between the two is the amount of starting materials and chemicals that are used. These include the peptide and buffers. The [^177^Lu]-LuCl_3_ used in the automated synthesis at our centre is NCA ^177^Lu. In contrast, the manual synthesis method reported in the literature (17) utilises ^177^Lu that contains chemical carriers. The implications of these added carriers are that larger amounts of DOTATATE peptide (up to 600 µg) are required during the synthesis to ensure that the required amounts of radiolabelled [^177^Lu]-DOTATATE is produced in the final product compared with approximately 200 µg using the NCA ^177^Lu. These extra amounts of the required peptide can contribute to the overall cost of the procedure.

The carrier-added ^177^Lu also has the disadvantage of containing additional impurities. During the production of the carried-added ^177^Lu by the ^176^Lu(n, γ)^177^Lu reaction via thermal neutron bombardment of enriched lutetium oxide some ^177m^Lu is also produced. ^177m^Lu has a long half-life (160.4 days) that will require the synthesis cassettes and other waste products, including potentially urine, to be stored for months to years prior to disposal (16).

As compared to the manual method, the automated synthesis system has two disadvantages: initial setup and ongoing consumable (cassettes only) costs. However this cost, in our opinion, is acceptable compared with the benefits that the automated system provides.

## Conclusions

The automated synthesis of [^177^Lu]-DOTATATE using Eckert & Ziegler Eurotope’s Modular-Lab Pharm Tracer® system is a robust, convenient and high yield approach to the radiolabelling of DOTATATE with large amounts of radioactive ^177^Lu, with almost negligible radiation exposure of the operators. The method we use also benefits from the use of no-carrier added ^177^Lu by reducing impurities, waste products and the mass of peptide required for adequate labelling. Much lower radiation doses to the operator is another significant advantage of the automated method. In our one year experience the system has performed reliably delivering the required dose for [^177^Lu]-DOTATATE therapy of patients with somatostatin-expressing neuroendocrine tumours.
